# Biological Activity Evaluation Against *Fusarium oxysporum*, *Fusarium circinatum,* and *Meloidogyne incognita* of Bioactives-Enriched Extracts of *Ruta graveolens* L.

**DOI:** 10.3390/molecules30102240

**Published:** 2025-05-21

**Authors:** Lorena Reyes-Vaquero, Elena Ibáñez, Soledad Sanz-Alférez, Gloria Nombela, Alma Angélica Del Villar-Martínez, Mónica Bueno

**Affiliations:** 1 Centro de Desarrollo de Productos Bióticos, Instituto Politécnico Nacional, Calle CEPROBI No. 8, Col. San Isidro, Yautepec 62731, Morelos, Mexico; lrvsaid@gmail.com (L.R.-V.); adelvillarm@ipn.mx (A.A.D.V.-M.); 2 Laboratory of Foodomics, Institute of Food Science Research, CIAL, CSIC, Nicolás Cabrera 9, 28049 Madrid, Spain; 3 Departamento de Biología, Facultad de Ciencias, Campus de Cantoblanco, Universidad Autónoma de Madrid, Calle Darwin 2, 28049 Madrid, Spain; soledad.sanz@uam.es; 4 Instituto de Ciencias Agrarias (ICA-CSIC), Departamento de Protección Vegetal, Serrano 115 Bis, 28006 Madrid, Spain; gnombela@ica.csic.es; 5 Laboratory for Aroma Analysis and Enology (LAAE), Department of Analytical Chemistry, Faculty of Sciences, Universidad de Zaragoza, Instituto Agroalimentario de Aragón-IA2 (Universidad de Zaragoza-CITA), c/ Pedro Cerbuna 12, 50009 Zaragoza, Spain

**Keywords:** antifungal activity, nematicidal activity, enriched extracts, pressurized liquid extraction, supercritical fluid extraction, bioactive compounds, *Ruta graveolens* L.

## Abstract

*Ruta graveolens* L. has been described as possessing antifungal and nematicidal activity. Among the bioactive compounds present in this plant, alkaloids and furanocoumarins have attracted considerable attention. The aim of this study was to evaluate the in vitro biological activity of extracts from rue enriched in bioactive compounds against *Fusarium oxysporum*, *F. circinatum,* and *Meloidogyne incognita*, and to correlate the chemical profile of the extracts with their biological activities. Six extracts with contrasting chemical profiles, obtained by pressurized liquid extraction and supercritical fluid extraction using green solvents, were selected for biological evaluation. The highest *F. oxysporum* growth inhibition was achieved with the extracts enriched in fatty acids and furanocoumarins at concentrations of 4, 8, and 16 mg/mL, while for *F. circinatum*, the highest growth inhibition was obtained using the extract enriched in terpenes at 16 mg/mL; moreover, the six extracts evaluated caused mortality in *M. incognita*. Therefore, enriched extracts of *R. graveolens* might be considered as an alternative for pathogen control on economically important crops such as potatoes, tomatoes, and onions, among others. Correlations between biological activities and chemical compositions suggest the importance of fatty acids against *F. oxysporum*, fatty acids and terpenes against *F. circinatum,* and alkaloids, coumarins, and furanocoumarins for *M. incognita*.

## 1. Introduction

Plants are affected by several diseases caused by microorganisms, such as bacteria, fungi, and nematodes, which cause a decrease in the quality and yield of crops [1]. *Fusarium oxysporum* is an important pathogen that causes vascular wilt in over a hundred plant species. *F. circinatum* is also an important pathogen causing the pitch canker disease in *Pinus* species and some other conifers [2]. Several species of *Fusarium oxysporum* are well represented among the communities of soilborne fungi, in all types of soil worldwide [3]. In general, this is considered a normal constituent of the fungal communities in the rhizosphere of plants [4]. All strains of *F. oxysporum* are saprophytic and able to grow and survive for long periods on organic matter in soil and in the rhizosphere of many plant species [5]. Furthermore, some strains of *F. oxysporum* are pathogenic to different plant species; they penetrate the roots, inducing either root rot or tracheomycosis when they invade the vascular system. Many other strains can penetrate roots, but do not invade the vascular system or cause disease [4]. The wilt-inducing strains of *F. oxysporum* are responsible for severe damage to many economically important plant species. *Fusarium* wilt pathogens exhibit a high level of host specificity and are classified into more than 120 *formae speciales* and races, based on the plant species and cultivars they infect [6].

Regarding plant-parasitic nematodes, they are a major threat to crop production systems all around the world [7] and, in some crops, nematodes are the dominant pathogen of any kind [8]. *Meloidogyne* is the most economically damaging genus since it causes root-knot disease, and *M. incognita* is extremely polyphagous, with a host range of up to 3000 plant species, affecting the production of many annual and perennial crops [9].

Usually, management of plant diseases caused by fungi or nematodes is carried out using chemicals, but the broad-spectrum biocides used to fumigate soil before planting, particularly methyl bromide, are environmentally damaging. Moreover, the uncontrolled or intensive use of fungicides or nematicides harms human health and induces resistance to these products in pathogenic microorganisms. Thus, their use is being restricted. The most cost-effective and environmentally safe control method is the use of resistant cultivars when these are available. In recent years, plant extracts have been highlighted as an alternative for plant disease control [10].

*Ruta graveolens* L., commonly known as rue, is an evergreen shrub of the Rutaceae family in the order of Sapindales [11]. Secondary metabolites such as furoquinolines, acridone alkaloids, furanocoumarins, coumarins, and terpenes have been identified in extracts from roots and aerial parts of this plant [12,13]. Essential oils obtained from rue by hydrodistillation have shown a nematicidal effect [14,15], while alkaloids and furanocoumarins extracted by sonication with ethyl acetate from leaves and roots of rue inhibited the growth of *Colletotrichum* spp., *Botrytis cinerea*, and *F. oxysporum* [16,17]. Mycelial growth inhibition of *Magnaporthe oryzae* by psoralen and bergapten, obtained through percolation, has also been reported [18].

*R. graveolens* extracts are commonly obtained by maceration [16], percolation [18], and sonication [12]. Nowadays, the need to develop efficient and sustainable extraction processes, which also comply with increasingly rigorous regulations, has led to the use of green technologies such as those based on compressed fluids [19]. Pressurized liquid extraction (PLE) and supercritical fluid extraction (SFE) can meet such requirements when using green solvents [20], such as ethanol, ethyl acetate, D-limonene, or CO_2_. The exploration of novel extraction techniques is crucial to discovering new bioactive compounds with potential applications in plant disease management. Traditional extraction methods often yield well-characterized extracts, but these new green extraction techniques can lead to the identification of previously undetected metabolites due to their ability to selectively extract molecules based on polarity, depending on the type of green compressed fluid or technology used [21]. Extracts obtained through PLE and SFE have shown promising insecticidal, antifungal, and nematicidal activities [22,23,24], supporting their use in sustainable agriculture. The search for new plant-derived extracts with unknown properties is fundamental for developing alternative disease control strategies, reducing the reliance on synthetic agrochemicals, and mitigating environmental risks.

Therefore, the present study aimed to evaluate the in vitro biological activity of *Ruta graveolens* L. extracts obtained by green compressed fluid techniques against *Fusarium oxysporum*, *F. circinatum,* and *Meloidogyne incognita*, correlating the chemical profile of enriched extracts with the biological activity.

## 2. Results

### 2.1. Biological Activity of Enriched Extracts of Ruta graveolens L.

#### 2.1.1. Selection of the Enriched Extracts for the Biological Activity Analysis

To evaluate the biological activity of extracts from the aerial parts and roots of *Ruta graveolens* L., six extracts with contrasting chemical profiles were selected (Table 1), based on the extraction protocols previously developed by Reyes-Vaquero et al. [25]. For PLE extracts, the extraction conditions were established using a 2^3^ factorial design and response surface methodology (RSM), which optimized the influence of temperature and solvent composition to enrich specific metabolite families. In the case of SFE, the selected extracts were obtained by performing sequential extractions on the same plant matrix, modifying pressure or sample humidity to target different compound groups. As a result, three extracts were obtained by PLE (PLE1 and PLE2 from aerial parts and PLE3 from roots) and another three by SFE collected in ethanol (SFE1 and SFE2 from aerial parts and SFE3 from roots). Differences in chromatographic profiles reflecting variations in the chemical composition of each extract can be seen in Supplementary Materials Figure S1, while tentative identification of compounds is provided in Table 2.

PLE1 is enriched in terpenes. It had the highest percentage of terpenes among the selected extracts (Table 1) and had the greatest relative abundance of terpenes and a high selectivity ratio T/(Al + FC + C) of 1.78 (see Table 3 of reference Reyes-Vaquero et al. [25]).

PLE2 possessed the best-balanced profile both in terms of relative abundance and percentage for terpenes, alkaloids, and furanocoumarins. This extract had the lowest difference from 33% (with a value of 14), showing that it is the best-balanced extract in these three families (see Table 3 of reference [25]). Table 1 shows that this extract is the one with a high presence (>20%) of the three families.

PLE3 had maximum amounts of relative abundance of alkaloids, furanocoumarins, and coumarins, and minimum of terpenes; therefore, the minimum selective ratio is T/(Al + FC + C) with a value of 0.05, as detailed in Table 3 of Reyes-Vaquero et al. [25]. In Table 1, this extract mainly stands out for the impact of alkaloids.

Regarding SFE extracts, SFE1 is enriched in fatty acids and furanocoumarins, SFE2 is enriched in furanocoumarins and alkaloids, and SFE3 is enriched in alkaloids, as shown in Table 1. These enrichments can be graphically depicted in Figures 4 and 5 of Reyes-Vaquero et al. [25].

**Figure 1:**
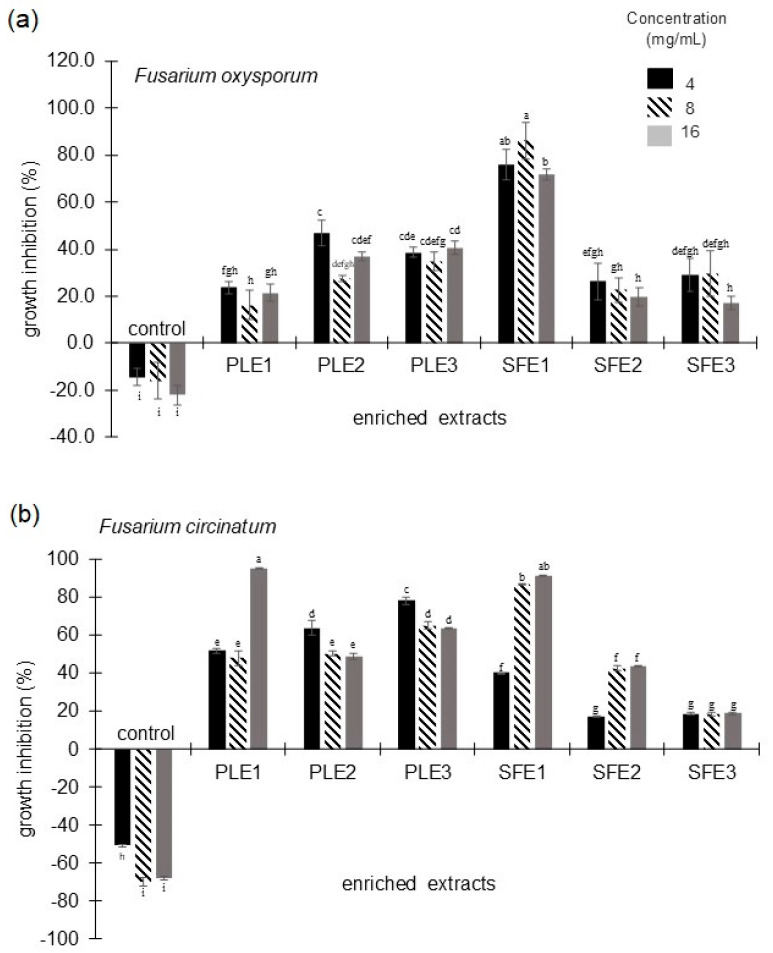
Antifungal activity of enriched extracts of *Ruta graveolens* L. Growth inhibition against (**a**) *Fusarium oxysporum* and (**b**) *Fusarium circinatum*. Control treatment consisted of PDB medium without extract. PLE1, PLE2, SFE1, and SFE2 obtained from aerial parts, and PLE3 and SFE3 from roots. Different letters within the same graphic indicate significant differences (*p* ≤ 0.05).

It must be taken into account that although the extracts PLE3 and SFE3 were enriched in alkaloids, their profile is different (Table 2 of this study and Figure 1 of reference [25]). In PLE3, fagarine and furofoline were exclusively identified, whereas in SFE3, the metabolites exclusively found were 3-methyl-2-nonyl-1H-quinolin-4-one and 2-methyl-3-undecyl-1H-quinolin-4-one. The other alkaloids were identified in both extracts but in different proportions, which can also affect the extracts’ bioactivity, as depicted in Figure 1 of reference [25].

#### 2.1.2. Antifungal Activity Against *Fusarium oxysporum* and *Fusarium circinatum*

Figure 1 shows the activity of the rue selected enriched extracts against *F. oxysporum* and *F. circinatum*. The extract enriched in fatty acids and furanocoumarins (SFE1) showed *F. oxysporum* growth inhibition (GI) higher than 70% with all tested concentrations. In contrast, with PLE1, SFE2, and SFE3 extracts, GI was below 30% (Figure 1a). The terpene-enriched extract (PLE1) and the one enriched in fatty acids and furanocoumarins (SFE1) showed the highest GI values (94.9 and 91.1%, respectively) against *F. circinatum*, with both showing this effect at a concentration of 16 mg/mL (Figure 1b). On the other hand, the lowest GI responses were observed with the extracts SFE2 at 4 mg/mL and SFE3 in the three concentrations.

#### 2.1.3. Nematicidal Activity Against *Meloidogyne incognita*

**Figure 2:**
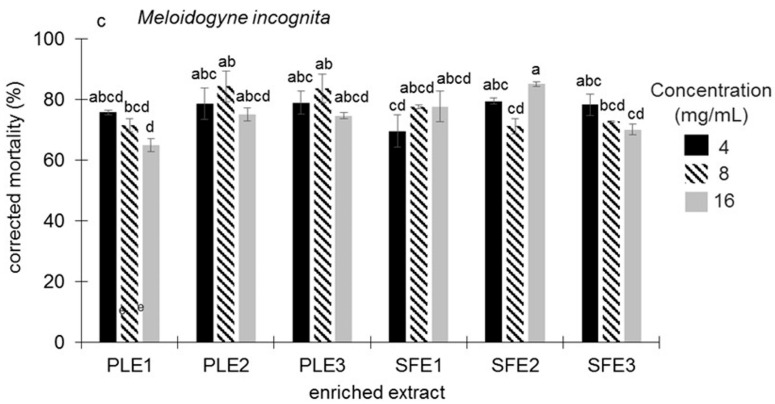
Nematicidal activity of enriched extracts of *Ruta graveolens* L. in terms of corrected mortality caused by *Meloidogyne incognita*. PLE1, PLE2, SFE1, and SFE2 obtained from aerial parts, and PLE3 and SFE3 from roots. Different letters within the same graphic indicate significant differences (*p* ≤ 0.05).

Regarding *Meloidogyne incognita*, the six enriched extracts of *Ruta graveolens* L. caused a corrected mortality (CM) of over 64%. The highest CMs were 85% with SFE2 at 16 mg/mL, and 84.3% with PLE2 and 83.7% with PLE3, with both extracts at 8 mg/mL (Figure 2). The lowest CM response was observed with the PLE1 extract at 16 mg/mL. These results show that rue is a plant that can be used for disease control in crops of agricultural interest.

#### 2.1.4. Biological Combined Action

As observed in Figure 1 and Figure 2, the studied extracts have biological activity against *F. oxysporum* and *F. circinatum* growth and caused *M. incognita* mortality. Since the ultimate goal of this study is to use these extracts as biopesticides, it is important to consider both the extraction yield and the concentrations tested. For this purpose, a quotient expressing the theoretical activity factor has been calculated for each activity together with a theoretical combined action (the sum of the three parameters), both parameters explained in Section 4.5.3. The corresponding values are presented in Table 3.

As an example, this parameter is illustrated using the three PLE extracts tested against *F. oxysporum* at 4 mg/mL (Figure 1a), with an extraction yield of 4.69, 2.25, and 12.06% for PLE1, PLE2, and PLE3, respectively. As the extraction yield for PLE1 is double that of PLE2, while the activities are half, this new parameter places both extracts on the same level in terms of functionality. On the other hand, comparing PLE2 with PLE3, the former shows a slightly better activity (47% vs. 39%), but in this case, the yield of PLE3 is six times higher, which makes this extract the most powerful in the example.

In other words, the higher this new parameter, the better the extract would be. Considering this, the best extract for *F. oxysporum* growth inhibition was SFE1; for *F. circinatum* growth inhibition, it was PLE3, and for *Meloidogyne incognita* mortality, it was SFE2. All of them were at 4 mg/mL, whereas the most complete in terms of bioactivity (theoretical combined action) was SFE1 at 4 mg/mL, followed by PLE3 and SFE2 at 4 mg/mL (the three extracts with values of this parameter above 5). However, it would be necessary to verify that activities can be additive.

### 2.2. Relationship Between Chemical Composition of the Selected Extracts and Biological Activity

In order to correlate biological activities with the chemical compositions of extracts, a Principal Component Analysis (PCA) was carried out. Activities obtained with concentrations of 4 mg/mL against *F. oxysporum*, 16 mg/mL against *F. circinatum,* and 8 mg/mL against *M. incognita* were selected for PCA because they showed the highest average activity among the six extracts.

The results of PCA showed that three principal components (PCs) describe 100% of the total data variability, divided into PC1 (54.71%), PC2 (31.68%), and PC3 (13.61%) terms.

**Figure 3:**
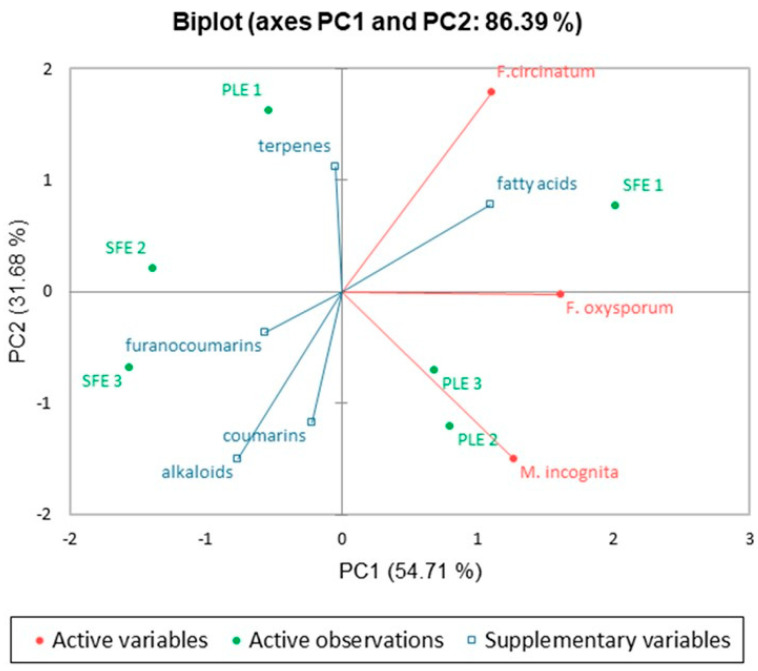
Principal Component Analysis (PCA) plot showing the projection of selected enriched extracts and their biological activities. The first two principal components explain 86.39% of the total data variability.

Figure 3 shows the distribution of the rue extracts according to biological activity in terms of PC1 and PC2 (86.39% of explained variance). Chemical profiles of enriched extracts are shown as supplementary variables to add more information to the plot.

## 3. Discussion

This study underscores the novelty of applying green compressed fluid extraction techniques (PLE and SFE) to obtain metabolite-enriched extracts from *Ruta graveolens* with distinct chemical profiles and, consequently, diverse bioactivities. Unlike traditional methods, these innovative approaches allow for the selective recovery of compounds with varying antifungal and nematicidal potency, depending on the metabolites extracted.

The differences in chemical composition resulting from each extraction method are key to understanding the observed biological activities. These variations are further explored through PCA, shown in Figure 3, which helps to visualize the relationships between metabolite families and their respective antifungal and nematicidal effects.

As shown in Figure 3, all biological activities have positive loadings on PC1, which means that this component arranges extracts according to their general ability to protect against antifungal and nematicidal activity. Therefore, SFE1 is the extract that shows the greatest protective effect, as also predicted in Table 3. It is suggested that the SFE1 extract, enriched in fatty acids and furanocoumarins, inhibits the growth of fungi *F. oxysporum* and *F. circinatum* and causes the mortality of *M. incognita*, because these metabolites act at different biological levels. It has been reported that furanocoumarins are compounds that react with DNA by intercalating pyrimidine bases in such a way that replication is interrupted [26], and that could be inhibiting the growth of microorganisms. While fatty acids affect the cell membrane intercalating through its components, disrupting the structure, and causing conformational changes [27].

From the point of view of the extract, SFE1 correlates with the antifungal activity against *F. oxysporum* (right part of PC1). Regarding the chemical profile, *F. oxysporum* is suggested to be inhibited thanks to the presence of fatty acids from aerial parts. This result agrees with a previous finding [12] in which an extract of aerial parts of rue, abundant in fatty acids, inhibited the growth of *F. oxysporum* in 71.5%. In that work, linolenic, oleic, palmitic, linoleic, and dodecanoic acids were identified as compounds responsible for the antifungal activity. All these compounds have been found in our compressed fluid extracts.

The second component of PCA (Figure 3) separates extracts according to their ability to inhibit the growth of *F. circinatum*, and on the other hand, the ability to cause the mortality of *M. incognita*.

The PCA shows that PLE1 and SFE1 extracts correlate with the antifungal activity against *F. circinatum* (upper part of PC2). This activity appears to be caused not only by fatty acids but also by terpenes, both compound families present in the aerial parts. In Reyes-Vaquero et al. [12], the activity against *F. circinatum* was not studied. Nevertheless, terpenes campesterol and phytol, also identified in that work, were suggested as important metabolites against antifungal activity for *F. oxysporum*, *F. proliferatum,* and *Stemphylium vesicarium*.

As for *M. incognita*, alkaloids seem to be the responsible compounds of the nematicidal activity, and coumarins and furanocoumarins are suggested to be related to a lesser extent.

Commented results are in agreement with those of Figure 1 and Figure 2 for the three activities, being obvious for the antifungal activities and more complex for the nematicidal activity. All the selected extracts have alkaloids and furanocoumarins in proportions above 10%, while coumarins are only present in the two root extracts and in proportions below 10%. Maybe, this is the reason why no differences in mortality of *M. incognita* have been appreciated in Figure 2. To confirm the hypothesis of a synergistic effect of these families against *M. incognita*, it would be necessary to test the alkaloids, furanocoumarins, and coumarins separately.

In previous reports, rue has shown nematicidal properties in vitro against *M. exigua* [28], *M. incognita* [14], *Xiphinema index* [29,30], and *Bursaphelenchus xylophilus* [15]. Furthermore, when used as green manure, rue leaves reduced the final population level of *Meloidogyne javanica* on sunflower [31] and controlled *X. index* on *Vitis vinifera* [32]. However, the chemical compounds responsible for these activities have not been identified. On the other hand, nematicidal activity of metabolites like furanocoumarins from parsley and terpenes from different Greek plants against *Meloidogyne* spp. has been reported [33,34].

Discussion of this section suggests that a combination of *Ruta graveolens* L. extracts SFE1 and PLE1 could provide a great inhibition of *F. oxysporum* and *F. circinatum* due to the presence of terpenes and fatty acids, while that against *M. incognita,* an extra enrichment on coumarins could be interesting in trying to reinforce the effect of alkaloids. All together, the results support the potential of *Ruta graveolens* extracts obtained through green extraction technologies as a sustainable and effective strategy for the control of key phytopathogens in agricultural production.

## 4. Materials and Methods

### 4.1. Samples of Ruta graveolens L.

Plants of *Ruta graveolens* L. were collected in the Campo experimental Emiliano Zapata, Yautepec, Morelos, México (18°49″ N to 99°05″ W, at 1064 masl), in January 2017. Taxonomic identification was performed by cross-checking a voucher specimen (No. 697262) in the collection of the National Herbarium of Mexico at the UNAM (MEXU). Samples were separated into aerial parts and roots. All samples were dried indoors at 25 °C to a constant weight, ground, sieved to a particle size of 500 µm, and stored in the dark [12].

### 4.2. Pressurized Liquid Extraction

Extractions were carried out in an accelerated solvent extractor (ASE-200, Dionex Corp., Sunnyvale, CA, USA) equipped with a solvent controller unit. For each extraction, aliquots of 1 g of dried aerial parts or roots were mixed with sea sand (0.25–0.30 mm diameter, Panreac Química) in a proportion of 1:1 (*w*/*w*). The mixture was placed into an 11 mL stainless-steel extraction cell. Extractions were performed by a static cycle of 20 min at a pressure of 103 bar.

Three PLE extracts were studied in this work, named PLE1, PLE2, and PLE3:

- PLE1 extract was obtained from the aerial part using D-limonene-ethyl acetate (50:50, *v*/*v*) as solvent at 40 °C;
- PLE2 was obtained from the aerial part using ethyl acetate (100%) as solvent at 105 °C;
- PLE3 was obtained from roots using ethyl acetate (100%) as solvent at 105 °C.

To obtain the amount of extract required for the activity assays, the PLE processes under the different conditions were repeated five times, and the extracts obtained under the same conditions were then combined, evaporated by a gentle steam of nitrogen (TurboVap^®^ LV Biotage, Uppsala, Sweden) and stored at 4 °C until analyses.

### 4.3. Supercritical Fluid Extraction

Supercritical CO_2_ (scCO_2_) extractions were performed in a homemade compressed fluid extractor coupled to a supercritical peltier CO_2_ pump PU–2080–CO2 from Jasco (Pfungstadt, Germany), which introduces CO_2_ into the extraction cell. Premier quality CO_2_ was used (Carburos Metálicos, Grupo Air Products, Madrid, Spain). Dried aerial parts or roots (1 g) were mixed with 1 g of sea sand and placed into an 8 mL stainless-steel extraction cell. Extractions were carried out at 40 °C, and the flow rate was 4 mL/min for 180 min. The complete extraction procedure was as follows: pressure was held at 100 bar for 60 min, then increased to 200 bar during the next 60 min, and finally to 350 bar during the last 60 min. Samples were moistened (water content 35% *w*/*w*) just before increasing the pressure to 350 bar. The extracts were collected in ethanol to avoid losses of volatile compounds.

Three SFE extracts were studied in this work, named SFE1, SFE2, and SFE3:

- SFE1 extract was obtained from the dry aerial parts extraction during the first 30 min at 200 bar;
- SFE2 was obtained from the moistened aerial parts sample during the last 60 min of the process at 350 bar;
- SFE3 was also obtained from the moistened sample at 350 bar, but this time only during the first 15 min from a sample of rue roots.

For each SFE process, extractions were performed in quintuplicate and subsequently pooled for each condition, evaporated by a gentle nitrogen stream, and stored at 4 °C until analyses.

### 4.4. Chromatography—Mass Spectrometry Analysis (GC–q-TOF-MS)

Rue extracts obtained by PLE and SFE were chemically characterized in a 7890B Agilent gas chromatograph (Agilent Technologies, Santa Clara, CA, USA) coupled to a 7200 quadrupole time-of-flight mass spectrometer (q-TOF-MS) (Agilent Technologies, Santa Clara, CA, USA) equipped with an electronic ionization (EI) source.

The GC oven method was performed following the procedure described previously [35]. Extended information for this procedure can be found elsewhere [25].

In order to facilitate the discussion, compounds were classified into families based on their chemical structure as detailed in Table 2: alkaloids (Al), terpenes (T), furanocoumarins (FC), fatty acids (FA), coumarins (C), phenolic compounds (Pc), amides (Am), ketones (K), and “others” (O) that do not correspond to the chemical structures previously mentioned.

### 4.5. Evaluation of Biological Activity Against Phytopathogenic Microorganisms

Six enriched extracts were selected based on the families of compounds they contained, in order to evaluate their biological activity. Enriched extracts were weighed and resuspended in distilled water, obtaining solutions at 4, 8, and 16 mg/mL. They were sterilized by filtration with an Acrodisc syringe filter, nylon membrane, 25 mm, 0.45 µm (Waters™ USA, Waters Corporation, Milford, MA, USA) [12].

#### 4.5.1. Antifungal Activity Against *Fusarium oxysporum* and *Fusarium circinatum*

*F. oxysporum* and *F. circinatum* strains were provided by Departamento de Biología, Universidad Autónoma de Madrid, Spain. The fungi were cultured on potato dextrose broth (PDB, Merck) at 25 ± 2 °C, in a 10:14 h light: dark regime during seven days. A spore suspension (2 × 10^4^ spores/mL) of each isolated fungus was prepared in PDB and used as the inoculum.

Bioassays were performed in 96-well, flat-bottom plates, through modified M38-A methodology for filamentous fungi [36]. Briefly, 100 μL of inoculum plus 100 μL of each tested extract concentration were added to each well. Plates were incubated at 28 °C, and fungal growth was evaluated by measuring absorbance at 540 nm every 24 h for five days, using a Synergy™ HT automated plate reader (BioTek™, Winooski, VT, USA). All treatments were analyzed in quadruplicate (analytical replicates). Growth inhibition was calculated using the formula previously reported [37] (Equation (1)):

(1) $$\mathrm{Growth}\;\mathrm{inhibition}\;\%=100\times (1-\frac{\mathrm{mean}\;\mathrm{absorbance}\;\mathrm{increase}\;\mathrm{experimental}\;\mathrm{wells}}{\mathrm{mean}\;\mathrm{absorbance}\;\mathrm{increase}\;\mathrm{control}\;\mathrm{wells}})$$

#### 4.5.2. Nematicidal Activity Against *Meloidogyne incognita*

A *Meloidogyne incognita* nematode population was obtained from infected roots of eggplant (*Solanum melongena*). Subsequently, the breeding was carried out in tomato plants (*Solanum lycopersicum* var. *Marmande*), in pot cultures in a growing chamber at 24 °C in the Instituto de Ciencias Agrarias (ICA-CSIC), in Madrid, Spain. Egg masses of nematodes were individually handpicked from infected tomato roots, placed in 24-well plates in distilled water, and incubated in the dark at 25 °C for 24 h to hatch eggs and allow the emergence of second-stage juveniles (J2).

Bioassays were performed in 96-well flat-bottom plates. To each well, 50 J2 nematodes suspended in 100 μL of distilled water plus 100 μL of each concentration (4, 8, and 16 mg/mL) of the enriched extracts were added. As a growth control, 50 J2 nematodes suspended in 200 μL of distilled water were used. Plates were covered to prevent evaporation and were maintained in the dark at 25 °C for 96 h. Then, the nematodes were washed to remove the extract, and 200 μL of distilled water was added to each well to confirm the mortality of the apparently dead nematodes. Plates were maintained in the dark at 25 °C for 24 h [38]. All treatments were analyzed in quadruplicate (analytical replicates). To identify dead nematodes, the plates were observed under a stereoscopic microscope (Leica MS5, Wetzlar, Germany), and nematodes that maintained mobility were quantified. Corrected mortality (%), eliminating natural death, was calculated according to the Schneider–Orelli formula [15] (Equation (2)):

(2) $$\mathrm{Corrected}\;\mathrm{mortality}\;\%=\frac{\left(\mathrm{mortality}\;\%\;\mathrm{in}\;\mathrm{treated}-\mathrm{mortality}\;\%\;\mathrm{in}\;\mathrm{control}\right)}{\left(100-\mathrm{mortality}\;\%\;\mathrm{in}\;\mathrm{control}\right)}\times 100$$

#### 4.5.3. Theoretical Activity Factor and Theoretical Combined Action

Theoretical activity factors were calculated taking into consideration not only the tested bioactivity but also the extraction yield and the tested extract concentration, following the quotient expressed in Equation (3). These factors were calculated for each activity along with a theoretical combined effect, calculated as the sum of the three parameters.

(3) $$\mathrm{Theoretical}\;\mathrm{activity}\;\mathrm{factor}\;=\frac{\mathrm{extraction}\;\mathrm{yield}\;\%}{\mathrm{tested}\;\mathrm{extract}\;\mathrm{concentration}}\times \mathrm{inhibition}\;\%\;\mathrm{or}\;\mathrm{mortality}\;\%\;$$

### 4.6. Statistical Analysis

Analysis of variance (one-way ANOVA) was performed on the chemical profile data in percentage. Fungal growth inhibition (%) and nematode corrected mortality (%) values were transformed to arcsine (P/100)^0.5^ for one-way ANOVA. ANOVA and post-hoc Tukey’s test for significant differences (*p* < 0.05) were carried out using InfoStat software version 2017 (Statistical software, 2017 version Córdoba, Argentina).

Principal Component Analysis (PCA) was applied to examine the correlation between antifungal and nematicidal activities and the selected extracts with the most distinct profiles, using the chemical profile of the enriched extracts as supplementary variables. The concentrations used for each activity corresponded to those with the highest average activity among the six extracts studied. PCA was performed using XLSTAT (2021.4.1 version).

## 5. Conclusions

Extracts enriched in bioactive metabolites from *Ruta graveolens* L. were successfully obtained using two different compressed fluid extraction technologies, PLE and SFE, employing environmentally friendly solvents.

Among the tested extracts, SFE1, enriched in fatty acids and furanocoumarins, showed the highest inhibitory effect against *Fusarium oxysporum* (>70%) and strong activity against *F. circinatum* (>90%), likely due to the synergistic action of its metabolites. PLE1, rich in terpenes, also exhibited high activity against *F. circinatum*. In nematode assays, all extracts caused >64% mortality in *Meloidogyne incognita*, with SFE2 (rich in alkaloids and furanocoumarins) being the most effective (85%).

Multivariate PCA analysis strongly supported the relationship between extract composition and bioactivity, particularly linking fatty acids and terpenes with antifungal effects, and alkaloids with nematicidal activity. Furthermore, when both extraction yield and bioefficacy were considered, SFE1 emerged as the most promising candidate.

These results highlight the value of enriched R. graveolens extracts as a promising and sustainable biotechnological tool for controlling major phytopathogens in economically important crops, thereby contributing to the reduction of chemical pesticide use.

## Figures and Tables

**Table 1:** Chemical profile in percentage of the chromatogram total area (excluding solvent) of selected enriched extracts of *Ruta graveolens* L (*n* = 5).

Group of Metabolites	PLE1	PLE2	PLE3	SFE1	SFE2	SFE3
Alkaloids (Al)	11.6 ± 0.7 ^D^	**23.8** ± 1.5 ^C^	**48.6** ± 3.2 ^A^	14.5 ± 3.8 ^D^	**41.7** ± 3.7 ^AB^	**40.4** ± 2.1 ^B^
Coumarins (C)	-	-	10.2 ± 0.5 ^A^	-	-	6.9 ± 0.2 ^B^
Furanocoumarins (FC)	21.9 ± 2.9 ^B^	**34.7** ± 4.5 ^A^	16.6 ± 1.6 ^C^	**23.5** ± 2.6 ^B^	**41.5** ± 2.6 ^A^	23.5 ± 3.1 ^B^
Terpenes (T)	**59.7** ± 3.8 ^A^	**30.2** ± 1.9 ^B^	3.5 ± 2.0 ^C^	4.6 ± 0.6 ^C^	0.8 ± 0.2 ^D^	4.3 ± 0.7 ^C^
Fatty acids (FA)	4.2 ± 0.3 ^D^	6.5 ± 0.5 ^C^	0.03 ± 0.02 ^E^	**52.6** ± 3.1 ^A^	7.6 ± 2.0 ^BC^	10.6 ± 1.5 ^B^
Amides (Am)	2.1 ± 1.2 ^B^	4.3 ± 3.1 ^AB^	9.2 ± 5.4 ^A^	1.3 ± 0.3 ^B^	1.6 ± 0.7 ^B^	2.0 ± 0.4 ^B^
Ketones (K)	-	-	-	0.2 ± 0.1 ^B^	0.1 ± 0.1 ^B^	1.7 ± 0.1 ^A^
Phenolic compounds (Pc)	-	-	-	0.2 ± 0.1 ^B^	0.4 ± 0.1 ^B^	5.8 ± 0.2 ^A^
Others (O)	0.5 ± 0.2 ^D^	0.6 ± 0.2 ^D^	11.8 ± 2.1 ^A^	3.2 ± 1.0 ^B^	6.4 ± 1.5 ^C^	4.7 ± 0.6 ^BC^

Those families in bold are relevant in each extract. Different letters in the same row indicate significant differences (*p* < 0.05).

**Table 2:** Compounds tentatively identified in the six different extracts of aerial parts or roots of Ruta graveolens L. obtained by PLE and SFE.

RT (min)	Tentative Identification	Match Factor	Monoisotopic Mass	PLE1	PLE2	PLE3	SFE1	SFE2	SFE3
	*Alkaloids*								
21.36	3-Methyl-2-nonyl-1H-quinolin-4-one	85	285.2093	−	−	−	−	−	+
22.16	Dictamnine	92	199.0633	−	−	+	+	+	+
23.60	2-Methyl-3-undecyl-1H-quinolin-4-one	83	313.2406	−	−	−	−	−	+
28.29	Fagarine	84	229.0739	+	+	+	+	+	−
28.94	Pteleine	88	229.0739	−	−	+	+	+	+
32.75	Skimmianine	92	259.0845	+	+	+	+	+	+
34.34	Kokusaginine	89	259.0845	+	+	+	+	+	+
34.66	1-Hydroxy-10-methyl-9(10H)-acridinone	90	225.0790	−	−	+	−	−	+
34.80	3-Methyl-2-undecyl-1H-quinolin-4-one	86	313.2406	−	−	+	−	−	+
37.87	1-Hydroxy-3-methoxy-10-methyl-9(10H)-acridinone	92	255.0895	−	−	+	−	−	+
40.03	Furofoline I	80	265.0739	−	−	+	−	−	−
40.17	Arborinine	90	285.1001	+	+	−	+	+	−
45.19	3-Methyl-2-pentyl-1H-quinolin-4-one	85	229.1467	−	−	+	−	−	+
47.15	Graveoline	80	279.0895	−	−	+	−	−	+
	*Furanocoumarins*								
19.63	Psoralen	92	186.0317	+	+	+	+	+	+
22.19	4-(1,1-Dimethylallyl)-9-methoxy-7H-furo[3,2-g][1]benzopyran-7-one	85	284.1049	−	−	−	−	−	+
24.06	Xanthotoxin	90	216.0423	−	−	+	−	−-	+
25.67	Bergapten	90	216.0423	+	+	+	+	+	+
28.03	Chalepensin	80	254.0943	+	+	+	+	+	+
29.39	Isopimpinellin	91	246.0528	−	−	+	+	+	+
35.82	Chalepin	82	314.1518	+	+	+	+	+	+
37.80	Rutamarin	90	356.1624	+	+	+	+	+	+
	*Terpenes*								
6.34	Sabinene	81	136.1252	−	−	+	−	−	−
6.40	*p*-Cymene	91	134.1096	−	−	+	−	−	−
6.76	*m*-Cymenene	97	132.0939	+	+	+	−	−	−
6.88	*p*-Mentha-1,5,8-triene	91	134.1096	−	−	+	−	−	−
7.22	*p*-Mentha-2,8-dien-1-ol	93	152.1201	+	+	+	−	−	−
7.24	*trans*-Verbenol	80	152.1201	+	+	−	−	−	−
7.28	Citronellal	86	154.1358	−	−	+	−	−	−
7.50	*p*-Mentha-1(7),8-dien-2-ol	84	152.1201	−	−	+	−	−	−
7.87	*trans*-3-Caren-2-ol	80	152.1201	+	+	−	−	−	−
7.92	*trans*-3(10)-Caren-2-ol	80	152.1201	+	+	−	−	−	−
8.04	*trans*-Carveol	93	152.1201	+	+	+	−	−	−
8.06	*trans*-2-Caren-4-ol	80	152.1201	+	+	−	−	−	−
8.07	Carveol	80	152.1201	+	+	−	−	−	−
8.15	Pulegone	80	152.1201	+	+	−	−	−	−
8.30	Carvenone	87	152.1201	+	+	−	−	−	−
8.49	*trans*-Ascaridolglycol	88	170.1307	+	−	+	−	−	−
9.98	Isoascaridol	80	168.1150	+	+	+	−	−	−
11.68	α-Ionone	80	192.1514	−	−	+	−	−	−
12.28	Calarene	81	204.1878	−	−	−	+	+	−
12.34	*trans*-β-Ionone	80	192.1514	−	−	−	−	−	+
14.48	α-Eudesmol	81	222.1984	+	+	−	−	−	−
22.48	Limonen-6-ol, pivalate	80	236.1776	+	+	−	−	−	−
26.40	Phytol	92	296.3079	+	+	−	+	+	−
43.13	Tocopherol	85	430.3811	+	+	−	+	+	−
45.29	Campesterol	88	400.3705	+	+	−	+	+	+
47.56	γ-Sitosterol	89	414.3862	+	+	−	+	+	+
	*Fatty acids*					−			
9.37	Nonanoic acid	80	158.1307	−	−	−	+	+	+
10.76	*n*-Decanoic acid	85	172.1463	−	−	−	+	+	−
11.82	2,5-Octadecadiynoic acid, methyl ester	82	290.2246	−	−	−	−	−	+
13.30	Fumaric acid, ethyl 2-methylallyl ester	82	198.0892	−	−	−	+	+	−
15.31	Dodecanoic acid	84	200.1776	−	−	+	+	+	+
18.44	Myristic acid	88	228.2089	−	−	−	+	+	+
21.94	Palmitic acid, methyl ester	80	270.2559	−	−	−	+	+	−
22.40	Palmitic acid	90	256.2402	+	+	−	+	+	+
23.94	Palmitic acid, ethyl ester	80	284.2715	−	−	−	+	+	+
25.51	8,11-Octadecadienoic acid, methyl ester	83	294.2559	−	−	−	+	+	−
26.51	Linoleic acid	87	280.2402	+	+	−	+	+	+
27.18	Oleic Acid	89	282.2559	−	−	−	+	+	−
27.25	Linolenic acid	88	280.2402	−	−	−	+	+	−
27.87	Stearic acid	86	284.2715	−	−	−	+	+	−
28.38	Linolenic acid, ethyl ester	80	306.2559	−	−	−	+	+	−
	*Coumarins*					−			
18.05	6,7,8-Trimethoxycoumarin	85	236.0685	−	−	−	−	−	+
18.17	7-Methoxycoumarin	89	176.0473	−	−	−	+	+	−
23.89	5,7-Dimethoxycoumarin	87	206.0579	−	−	−	−	−	+
25.32	Seselin	80	228.0786	−	−	+	−	−	−
27.05	Ostol	87	244.1099	−	−	+	−	−	+
31.34	5-Hydroxy-7-methoxy-2-methyl-6-(3-methyl-2-butenyl)- chromone	83	274.1205	−	−	−	−	−	+
32.46	3-(1,1-dimethylallyl) scopoletin	87	260.1049	−	−	+	−	−	+
	*Phenolic compounds*								
8.18	1,3-bis(1,1-dimethylethyl)-benzene	80	190.1722	−	−	−	−	−	+
11.46	4-Tert-butyl-2-(2-methylbutan-2-yl)phenol	83	220.1827	−	−	+	−	−	−
14.55	2,4-Di-tert-butylphenol	83	206.1671	−	−	−	+	+	+
18.26	Turmeronol A	80	232.1463	+	+	−	+	+	+
18.33	€-Coniferyl alcohol	83	180.0786	−	−	−	−	−	+
20.27	Isogentisin	80	258.0528	+	+	−	−	−	−
	*Amides*								
21.47	Myristamide	84	227.2249	−	−	+	−	−	−
33.04	Oleamide	90	281.2719	+	+	+	+	+	+
	*Ketones*								
9.79	2-Undecanone	80	170.1671	−	−	−	+	+	+
13.42	2-Tridecanone	85	198.1984	−	−	−	+	+	+
	*Others*								
7.40	Myrtenyl methyl ether	80	166.1358	−	−	+	−	−	−
10.39	Piperonal	80	150.0317	−	−	−	−	−	+
10.55	Tricycloekasantalal	85	178.1358	−	−	−	+	+	+
10.61	7-Tetradecene	83	196.2191	−	−	−	+	+	−
11.09	1,9-Nonanediol	87	160.1463	−	−	−	+	+	−
11.78	Tricyclo[4.4.1.1(3,8)]dodeca-4,9-diene	84	160.1252	−	−	−	+	+	−
11.97	2,6,10-Trimethyltetradecane	81	240.2817	−	−	−	+	+	−
14.35	Heptacosane	80	380.4382	+	+	−	+	+	+
14.41	β-Acorenol	80	222.1984	−	−	−	+	+	−
14.99	5,6,7,7a-tetrahydro-4,4,7a-trimethyl-2(4H)-Benzofuranone	80	180.1150	−	−	−	+	+	+
15.41	Syringaldehyde	82	182.0579	−	−	−	+	+	−
15.80	cis,α-Santalol	81	234.1984	+	+	−	−	−	−
16.19	2,5,5,8a-Tetramethyl-6,7,8,8a-tetrahydro-5H-naphthalen-1-one	83	204.1514	−	−	−	−	−	+
16.28	Illudol	80	252.1725	−	−	−	+	+	−
17.41	2-Hexadecanol	83	242.2610	−	−	−	+	+	−
18.57	Santalcamphor	84	236.1776	+	+	−	+	+	−
18.69	6-Hydroxy-4,4,7a-trimethyl-5,6,7,7a-tetrahydro2(4H)benzofuran	83	196.1099	−	−	−	+	+	−
19.96	Heptadecane-2,4-dione	82	268.2402	+	+	−	−	−	−
21.29	7-Methyl-Z-tetradecen-1-ol acetate	80	268.2402	−	−	−	+	+	−
22.05	11,13-Dimethyl-12-tetradecen-1-ol acetate	81	282.2559	−	−	−	+	+	−
23.76	Tetratetracontane	85	618.7043	−	−	−	+	+	−
24.95	α-Tocospiro A	82	462.3709	−	−	−	+	+	−
41.95	3,4-bis(1,3-benzodioxol-5-ylmethyl)dihydro-(3R-trans)-2(3H)-Furanone	83	354.1103	−	−	+	−	−	+

RT: retention time, PLE1: obtained from the aerial part using D-limonene-ethyl acetate (50:50, *v*/*v*) at 40 °C; PLE2: obtained from the aerial part using ethyl acetate (100%) at 105 °C; PLE3: obtained from roots using ethyl acetate (100%) at 105 °C; SFE1: obtained from the dry aerial parts extraction during the first 30 min at 200 bar; SFE2: obtained from the moistened aerial parts sample the last 60 min of the extraction process at 350 bar; SFE3: obtained from the moistened sample at 350 bar the first 15 min from a sample of rue roots; − absence; + presence. Main fragments of the tentatively identified compounds and differential relative abundance of the extracts can be found in Reyes-Vaquero et al. [25].

**Table 3:** Theoretical activity factors for the growth inhibition of *F. oxysporum* and *F. circinatum* and the corrected mortality of *M. incognita,* taking into consideration the extraction yield and the tested extract concentration of diverse enriched extracts obtained from *R. graveolens*. Theoretical combined action of the three biological activities.

Extract	*Fusarium oxysporum*			*Fusarium circinatum*			*Meloidogyne incognita*			Theoretical Combined Action		
	Concentration (mg/mL)											
	4	8	16	4	8	16	4	8	16	4	8	16
PLE1	0.28 ± 0.02	0.09 ± 0.02	0.06 ± 0.01	0.61 ± 0.01	0.28 ± 0.01	0.28 ± 0.00	0.89 ± 0.01	0.42 ± 0.01	0.19 ± 0.01	1.77 ± 0.02	0.79 ± 0.02	0.53 ± 0.01
PLE2	0.26 ± 0.02	0.08 ± 0.01	0.05 ± 0.01	0.36 ± 0.01	0.14 ± 0.03	0.07 ± 0.00	0.44 ± 0.01	0.24 ± 0.01	0.11 ± 0.01	1.06 ± 0.03	0.46 ± 0.01	0.23 ± 0.01
PLE3	1.16 ± 0.03	0.52 ± 0.03	0.31 ± 0.01	**2.35 ± 0.03**	0.98 ± 0.01	0.48 ± 0.01	2.38 ± 0.04	1.26 ± 0.03	0.56 ± 0.01	**5.89 ± 0.02**	2.76 ± 0.03	1.35 ± 0.01
SFE1	**2.44 ± 0.10**	1.39 ± 0.06	0.58 ± 0.01	1.29 ± 0.01	1.40 ± 0.01	0.73 ± 0.01	2.24 ± 0.07	1.25 ± 0.01	0.62 ± 0.01	**5.97 ± 0.13**	4.03 ± 0.07	1.93 ± 0.01
SFE2	1.10 ± 0.16	0.47 ± 0.06	0.21 ± 0.01	0.71 ± 0.01	0.88 ± 0.02	0.45 ± 0.00	**3.30 ± 0.02**	1.48 ± 0.02	0.88 ± 0.01	**5.10 ± 0.19**	2.83 ± 0.06	1.54 ± 0.02
SFE3	0.57 ± 0.07	0.29 ± 0.05	0.08 ± 0.01	0.36 ± 0.01	0.18 ± 0.01	0.09 ± 0.00	1.53 ± 0.03	0.71 ± 0.01	0.34 ± 0.01	2.46 ± 0.08	1.18 ± 0.04	0.52 ± 0.01

Those families in bold are relevant in each extract.

## Data Availability

The original contributions presented in this study are included in the article and Supplementary Material.

## Supplementary Materials

The following supporting information can be downloaded at https://www.mdpi.com/article/10.3390/molecules30102240/s1, Figure S1: Total ion current (TIC) chromatograms obtained by gas chromatography-mass spectrometry (GC-MS) for six different extracts of *Ruta graveolens* L. Differences in chromatographic profiles reflect variations in the chemical composition of each extract. More compositional details are provided by Reyes Vaquero et al. [25]. Table S1. Lethal doses (LD50) of enriched extracts from *Ruta graveolens* L. required to inhibit 50% of the growth of *Fusarium oxysporum* (FO), *Fusarium circinatum* (FC), and *Meloidogyne incognita* (MI).

## Abbreviations

The following abbreviations are used in this manuscript:

Al	Alkaloids
Am	Amides
ANOVA	ANalysis Of VAriance
CM	Corrected Mortality
C	Coumarins
FA	Fatty Acids
FC	FuranoCoumarins
GC–q-TOF-MS	Gas Chromatography—Mass Spectrometry analysis
GI	Growth Inhibition
J2	Second-Stage Juveniles
K	Ketones
O	Other compounds
Pc	Phenolic compounds
PLE	Pressurized Liquid Extraction
PCA	Principal Component Analysis
PC	Principal Component
scCO_2_	Supercritical CO_2_
SFE	Supercritical Fluid Extraction
T	Terpenes
