# Supplementary material for: Biological Activity Evaluation Against Fusarium oxysporum, Fusarium circinatum, and Meloidogyne incognita of Bioactives-Enriched Extracts of Ruta graveolens L
Source: Molecules. 2025 May 21;30(10):2240. doi: 10.3390/molecules30102240 (PMC12113832; doi:10.3390/molecules30102240)
Supplement: Supplementary file 1 [file molecules-30-02240-s001.zip › molecules-3613147-supplementary.pdf]

Supplementary Materials

Biological Activity Evaluation Against *Fusarium oxysporum*, *Fusarium circinatum*, and *Meloidogyne incognita* of Bioactives-Enriched Extracts of *Ruta graveolens* L.

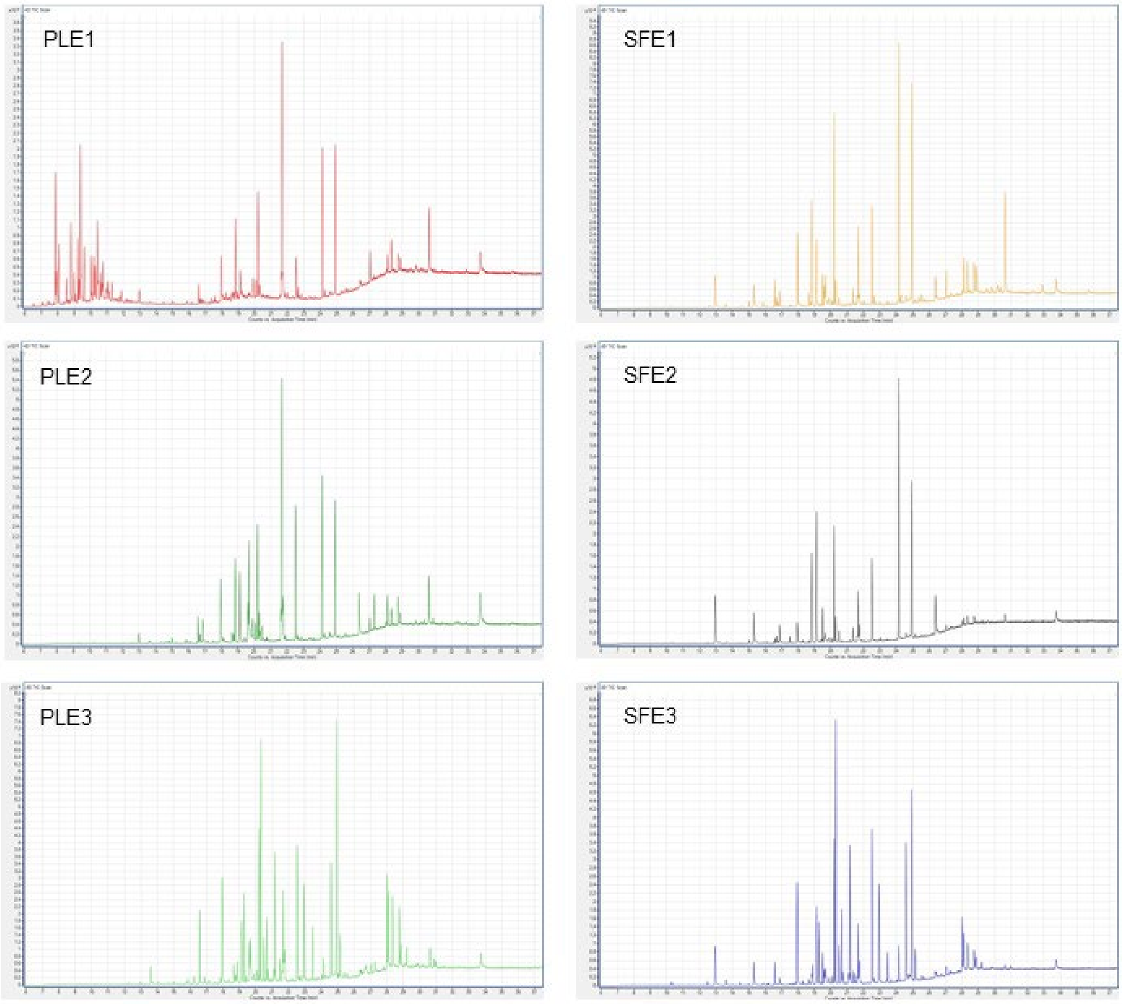

Figure S1. Total ion current (TIC) chromatograms obtained by gas chromatography-mass spectrometry (GC-MS) for six different extracts of *Ruta graveolens* L. Differences in chromatographic profiles reflect variations in the chemical composition of each extract. More compositional details in Reyes-Vaquero et al [25].

Table S1. Lethal doses (LD50) of enriched extracts from *Ruta graveolens* L. required to inhibit 50% of the growth of *Fusarium oxysporum* (FO), *Fusarium circinatum* (FC) and *Meloidogyne incognita* (MI).

Enriched extracts	Microorganism	LC_{50}
PLE1	FO	110.77
	FC	5.07
	MI	13.07
PLE2	FO	22.60
	FC	11.59
	MI	4.91
PLE3	FO	79.27
	FC	4.96
	MI	2.79
SFE1	FO	2.68
	FC	4.28
	MI	1.30
SFE2	FO	71.50
	FC	69.12
	MI	1.61
SFE3	FO	806.04
	FC	1618.98
	MI	2.76
